# Developmental origins of disease highlight the immediate need for expanded access to comprehensive prenatal care

**DOI:** 10.3389/fpubh.2022.1021901

**Published:** 2022-11-24

**Authors:** Chloe R. McDonald, Andrea M. Weckman, Julie K. Wright, Andrea L. Conroy, Kevin C. Kain

**Affiliations:** ^1^Sandra A. Rotman (SAR) Laboratories, Sandra Rotman Centre for Global Health, University Health Network-Toronto General Hospital, Toronto, ON, Canada; ^2^Faculty of Medicine, University of Toronto, Toronto, ON, Canada; ^3^Department of Pediatrics, School of Medicine, Indiana University, Indianapolis, IN, United States; ^4^Toronto General Hospital Research Institute, University Health Network, Toronto, ON, Canada; ^5^Tropical Disease Unit, Division of Infectious Diseases, Department of Medicine, University of Toronto, Toronto, ON, Canada

**Keywords:** pregnancy, *in utero* development, maternal health, antenatal care, origins of disease

## Abstract

The prenatal environment plays a critical role in shaping fetal development and ultimately the long-term health of the child. Here, we present data linking prenatal health, *via* maternal nutrition, comorbidities in pregnancy (e.g., diabetes, hypertension), and infectious and inflammatory exposures, to lifelong health through the developmental origins of disease framework. It is well-established that poor maternal health puts a child at risk for adverse outcomes in the first 1,000 days of life, yet the full health impact of the *in utero* environment is not confined to this narrow window. The developmental origins of disease framework identifies cognitive, neuropsychiatric, metabolic and cardiovascular disorders, and chronic diseases in childhood and adulthood that have their genesis in prenatal life. This perspective highlights the enormous public health implications for millions of pregnancies where maternal care, and therefore maternal health and fetal health, is lacking. Despite near universal agreement that access to antenatal care is a priority to protect the health of women and children in the first 1,000 days of life, insufficient progress has been achieved. Instead, in some regions there has been a political shift toward deprioritizing maternal health, which will further negatively impact the health and safety of pregnant people and their children across the lifespan. In this article we argue that the lifelong health impact attributed to the perinatal environment justifies policies aimed at improving access to comprehensive antenatal care globally.

## Introduction

**Figure 1:**
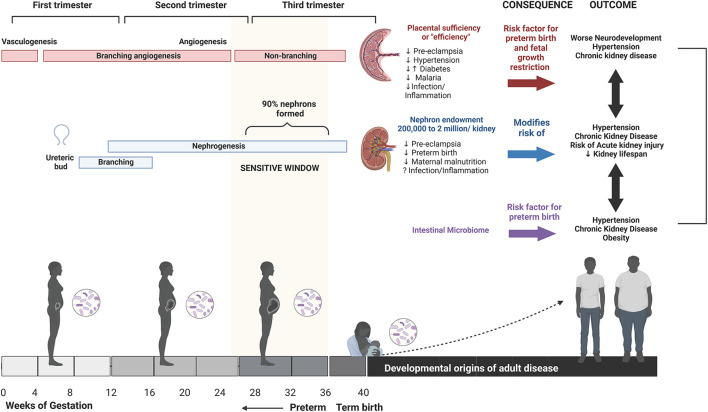
Developmental origins of health and disease. From the time of conception, and throughout gestation, the health of the mother and resulting changes in the *in utero* environment can impact the health and development of the child and last well into adulthood. Maternal health, including stress, nutritional status, metabolic disorders, infection, and inflammation during pregnancy, can impact *in utero* development *via* multiple pathways including altered placental vascular development, nephrogenesis, and microbiome. This can result in an increased risk of poor health outcomes in the child, including a potential impact on neurodevelopmental processes, or may manifest in adulthood as an increased risk of metabolic disorders, cardiovascular disorders, or acute kidney injury. Created using Biorender.com.

The first 1,000 days of life, from conception to 1 years of age, is a period of incredible vulnerability that can dramatically alter the life trajectory of a child (1, 2). From the time of conception, the developmental course of the embryo, and subsequently the fetus, is dependent on the health of the mother. Pre- and post-natal care policies that promote maternal health have the potential to shape a child's path to survive and thrive with intergenerational benefits. Most programs focusing on the first 1,000 days promote early childhood interventions that support children in reaching their developmental milestones (1). However, taking a view of a person's lifelong health trajectory through the lens of the developmental origins of health and disease framework identifies a broader scope of impact for maternal health interventions (Figure 1). A growing body of evidence has documented the impact of the perinatal environment on lifelong health. Diverse prenatal exposures can impact fetal growth and development *in utero* and alter long-term health outcomes. These include environmental exposures, medications, maternal nutrition, comorbidities in pregnancy including gestational diabetes and hypertension, and infectious and inflammatory exposures (3–6). During pregnancy, early patterning decisions followed by exquisitely regulated developmental processes are required for healthy fetal growth and development (7). While some disturbances in these carefully regulated processes may lead to fetal demise and pregnancy loss, other non-lethal perturbations may subtly alter organ formation and maturation. The consequences of these alterations may manifest later in life during periods of rapid growth and development (e.g., adolescence), or into adulthood, leading to the unmasking of health problems or premature onset of non-communicable diseases (7).

As evidence documents the impact of the perinatal environment on lifelong health, there is a need to translate this evidence into policy that promotes comprehensive and accessible antenatal care (ANC) and allied reproductive and pediatric healthcare. Here, we present evidence of developmental origins of disease and propose that enhanced access to ANC is a crucial pathway to improve prenatal health, healthy *in utero* development, and healthy outcomes across the lifespan. At present there are no globally uniform standards for the provision of ANC. In 2002, the WHO recommended focused antenatal care (FANC), which suggests a minimum of four scheduled visits for uncomplicated pregnancies (8). After an evidence-based policy review in 2015 showed increased risk for perinatal death with FANC compared to eight-visit models, the WHO updated its minimum recommended number of ANC visits from four to eight (9). Despite the WHO's updated recommendations, FANC, or modified versions, remains the most common model of ANC, based on widespread use in low- and middle-income countries (LMIC). Lack of consistency around ANC policies and recommendations, combined with shifting healthcare priorities and limited resources, leaves far too many pregnant people and children at risk. Recognizing the long-term impact of exposures in pregnancy on human health and wellbeing, there is an urgent need to prioritize policies around maternal health and pregnancy care. We propose that the developmental origins of disease framework provides even more evidence for the need to protect health at this vulnerable time.

## Evidence for developmental origins of disease

### Maternal health, adverse birth outcomes, and poor health trajectory

Multiple cardiovascular, metabolic, immune, and neurological morbidities have been linked to developmental, or *in utero*, origins (4, 5, 10). Among the most direct consequences of compromised *in utero* development are adverse pregnancy outcomes such as preterm birth (PTB; birth prior to 37 completed gestational weeks of pregnancy), being born with low birth weight (LBW; birth weight <2,500 g), or small-for-gestational age (SGA; birth weight below the 10th percentile for gestational age). Each of these adverse pregnancy outcomes confers an excess risk of morbidity and mortality in the immediate period and throughout the lifespan. PTB is now the leading cause of neonatal mortality worldwide, and a leading cause of death in children under five (11). In addition to perinatal mortality, multiple long-term health outcomes are associated with PTB including neurodevelopmental disorders as well as cardiovascular and metabolic disease in adulthood (12–14). An estimated 27% of all births in LMIC are SGA as a result of intrauterine growth restriction (15). Like PTB, SGA is associated with a greatly increased risk of neonatal morbidity and mortality and can have lifelong consequences including growth delay, neurodevelopmental impairment, and cerebral palsy (16, 17). The risk of PTB, LBW, and SGA is closely tied to maternal health and multiple factors including maternal malnutrition, exposure to infectious diseases, or maternal metabolic disorders, increase the risk of poor birth outcomes. The role of identifiable birth outcomes, such as PTB and LBW, on developmental origins of disease is more easily quantifiable, as the association between adverse birth outcomes and childhood health is well-established. However, research is demonstrating childhood and lifelong health outcomes associated with maternal health, in the absence identifiable poor birth outcomes (Table 1).

**Table 1:** Maternal exposures during pregnancy that may impact long-term health outcomes and potential interventions.

**Conditions**	**Examples**	**Long-term outcomes**	**Reference examples**	**Interventions to include in antenatal care**
**Maternal exposures**				
Malnutrition	Micronutrient and/or macronutrient deficiencies, obesity	Type 2 diabetes, obesity, coronary artery and other cardiovascular disease, early cardiovascular death, abnormal adiposity, epigenetic and neurodevelopmental programming, neurocognitive and neuropsychiatric conditions	(20–22, 28–37)	Micronutrient/macronutrient supplementation; nutritional screening, counseling, and support
Hypoxia	Smoking, high altitude, air pollution, maternal respiratory disease, fetal hypoxia due to obstetric complications	Obesity, cardiovascular disease, structural brain changes, neurocognitive, and neuropsychiatric disorders	(22, 119–121)	Support for smoking cessation; access to masks; skilled providers at delivery and access to emergency obstetric care
Infections	Influenza, SARS-CoV-2, urinary tract infections, sexually transmitted infections, malaria, zika virus	Structural brain changes, epigenetic and neurodevelopmental reprogramming, neurocognitive and neuropsychiatric conditions, cardiometabolic outcomes (e.g., increased BMI and blood pressure)	(3, 18, 19, 122–126)	Vaccination (e.g., influenza, SARS-CoV-2); insecticide treated nets, vector control, and malaria chemoprevention; comprehensive screening and treatment for genitourinary tract infections
Stress	Prenatal maternal anxiety, depression, elevated glucocorticoids, exposure to natural disasters or pandemics	Epigenetic and neurodevelopmental reprogramming, neurocognitive and neuropsychiatric outcome, cardiometabolic outcomes (e.g., obesity, cardiovascular disease, hypertension)	(20–22, 24–27, 127)	Stress resilience programs; mental health supports; screening and treatment for maternal stress
Maternal microbiome	Intestinal dysbiosis (maternal/fetal), mode of delivery, early antibiotic exposure	Asthma, impaired immune development, obesity, neurodevelopmental and neuropsychiatric outcomes	(50, 52, 53, 66, 128)	Prevention of factors that alter maternal microbiota (e.g., stress, gestational diabetes, malnutrition); antibiotic stewardship; microbiome optimization (e.g., pre and probiotic supplementation for those at risk for dysbiosis)
**Maternal comorbid conditions**				
Hypertension	Pre-existing hypertension, preeclampsia, HELLP	Cardiovascular disease, neurodevelopmental and behavioral outcomes	(22, 129, 130)	Effective blood pressure control; early diagnosis (screening) and treatment
Diabetes	Gestational diabetes, type 2 diabetes, type I diabetes	Altered postnatal growth trajectory, early onset cardiovascular disease, neurobehavioral abnormalities, neuropsychiatric disorders	(22, 131–134)	Screening for gestational diabetes; effective glucose management; hospital-based delivery
Chronic infections	HIV infection, viral hepatitis	Increased risk for gestational diabetes; mother-to-child transmission; neurodevelopmental and neurocognitive outcomes,	(135–137)	Effective anti-retroviral access and use in pregnancy; maternal screening; infant treatment and follow-up
**Birth Outcomes**	
Prematurity “Born too soon”		Hypertension, elevated LDL, diabetes, chronic lung disease, vision problems, chronic kidney disease	(12–14, 138, 139)	Prevention of the above maternal exposures and comorbidities; early and consistent access to ANC; access to sexual education and family planning
Fetal growth restriction/small for gestational age “Born too small”		Coronary heart disease, type 2 diabetes, chronic kidney disease, neurological and cognitive outcomes	(4, 16, 17, 140, 141)	Prevention of the above maternal exposures and comorbidities; early and consistent access to ANC; access to sexual education and family planning

### The developmental origins of neurocognitive and neuropsychiatric disorders

Multiple studies have linked maternal infection with poor neurocognitive outcome and with neuropsychiatric disorders in children (3, 18). Prenatal infection or inflammatory disorders are associated with an increased risk of schizophrenia, autism spectrum disorder, and depression in exposed children. This occurs in the absence of congenital infection and has been linked to the impact of maternal immune activation on fetal neurodevelopment. For example, malaria in pregnancy, one of the most common maternal infections globally, has been associated with impaired language development in exposed offspring (19).

Maternal stress is also associated with the development of neuropsychiatric disorders later in life (20–23). Maternal anxiety and/or depression has been linked with an increased risk of childhood emotional and behavioral problems in early adulthood, independent of postpartum symptoms or socioeconomic factors (24). Children born to mothers who experienced maternal anxiety have demonstrated epigenetic age acceleration at 10 years of age (24). Imaging studies have associated prenatal maternal distress with structural changes in the infant brain, specifically hippocampal connectivity and cortical morphology (25, 26). Investigators have begun to propose early interventions to teach and facilitate stress management for at-risk mothers (27).

### The developmental origins of metabolic disorders and cardiovascular disease

Development of metabolic disorders such as type 2 diabetes and cardiovascular disease have been linked to maternal nutritional status (28). Maternal undernutrition is often associated with intrauterine growth restriction, which increases the risk of type 2 diabetes and cardiovascular disease in offspring in later life. Although a large proportion of children whose mothers experience nutritional deficiencies will not show a birth phenotype, fetal hormonal adaptations and epigenetic changes are evident. Maternal obesity is also a risk factor for metabolic and cardiovascular disorders in children (29). Non-communicable diseases are rising globally with the fastest rates in LMIC, where weak health systems make it difficult to provide adequate care for people living with chronic conditions. This results in more women at risk of malnutrition in pregnancy and an increasing number of children at risk.

Hypotheses around the mechanisms underlying developmental origins of disease propose that as the fetus aims to optimize growth in response to energy and nutrient availability *in utero*, fetal physiology undergoes structural and functional adaptations that persist after birth. Therefore, in conditions of nutrient deficiency, the fetus adapts to prioritize glucose and lipid storage (30). However, when the post-natal environment is nutritionally abundant, these *in utero* adaptations become maladaptive and can contribute to the development of metabolic syndromes and cardiovascular disorders in later life. Several studies have established an association between individuals exposed to maternal nutrient deprivation *in utero*, and risk of LBW, obesity and cardiovascular disease in adulthood (30–32). In particular, individuals born LBW who demonstrate compensatory “catch up” growth in infancy and early childhood appear to have increased risk of adverse cardiovascular outcomes in adulthood. Maternal overweight, obesity, and increased gestational weight gain, are associated with an increased risk of large-for-gestational-age newborns and abnormal adiposity (33–35). Despite differences in birth phenotype compared to children of undernourished women, these offspring are also at increased risk of obesity, metabolic syndrome in childhood (36) and adulthood (37), and of early cardiovascular death.

## Mechanisms of action

### The *in utero* environment and early development

The *in utero* environment plays a critical role in a child's post-natal health, behavior, and long-term development but remains a neglected area of research, due at least in part, to a lack of prioritization of maternal health and the inherent risks and challenges associated with conducting research in pregnancy. From conception through to delivery, each stage of *in utero* development can have profound implications on the healthy growth and development of the child. Full reviews of *in utero* development have been reported in detail (38, 39). After an initial period of embryonic development, fetal development begins at 8 weeks of gestation. The first 12 weeks of fetal development are marked by formation of all major organ systems, which are established by 12 weeks with ongoing maturation and development through to term and into childhood. In the second trimester, essential processes in lung development are initiated, as well as the formation of hearing and facial features. Throughout the third trimester numerous maturation processes occur, including formation of muscle and fat stores, until the fetus is considered to be at term by 37 completed weeks of gestation. Across embryonic and fetal development, central nervous system development proceeds by sequential periods of neurogenesis, cell migration, synapse formation, and synaptic pruning. Functional neural circuitry undergoes essential periods of development in the third trimester. Each of these developmental periods require an orchestrated and tightly regulated set of initiation and completion signals. Given the unique developmental events occurring within each trimester, the timing of adverse maternal health events such as an infection in pregnancy could have varying impacts on fetal health based on gestational age. Alternatively, conditions such as sustained maternal malnutrition, have the potential for cumulative impact on the *in utero* environment. Collectively, these data reveal that disruption to the tightly regulated *in utero* environment modified early developmental processes (6, 21). Thus the nature and timing of events that affect maternal health and modify the intrauterine environment have the potential to disrupt essential and tightly regulated processes of embryonic and fetal development.

Poor maternal health and resulting changes to the *in utero* environment have been associated with altered fetal organ structure, epigenetic modification of gene expression, regulation of cellular aging, and the composition of the microbiome (5) (Table 1). For example, three processes that begin *in utero* and contribute to life-long human health are placental vascular development, fetal nephrogenesis, and the establishment of the baby's microbiome.

### Development of the placenta

Fetal development is dependent on the growth, structure, and function of the placental vasculature. By term, placental vasculature is over 500 km in length and 15 m^2^ in surface area (40). Several prominent pregnancy-related pathologies including gestational diabetes mellitus, intrauterine growth restriction, and pre-eclampsia have been linked to placental vasculature dysfunctions including inadequate perfusion, and villous morphological alterations and infarction (41–43). The placenta is formed through sequential processes of vasculogenesis (the formation of new vasculature) and angiogenesis (refinement of existing blood vessels). Once established, the placental vasculature undergoes processes of transformation (branching and elongation of capillaries) and maturation of vessels throughout gestation. The rate of capillary elongation increases dramatically beginning at gestational week 25 and total length increases at an exponential rate until term (44). The accelerated angiogenic processes occurring in the third trimester mirror and support the rapid fetal growth occurring at this time. Placental pathologies are closely tied to maternal health and therefore provide a potential mechanistic pathway for developmental origins of disease (10).

### Nephrogenesis

Similar to the ordered processes that characterize placental development, early kidney development involves sequential steps of ureteric bud outgrowth followed by ureteric branching and branching nephrogenesis. Nephrogenesis begins at 5 weeks of gestation and is completed by 36 weeks of gestation, so an individual is born with their entire complement of nephrons or “nephron endowment.” Nephron endowment in humans ranges from 200,000 to 2 million nephrons per kidney. An estimated 90% of nephrons are formed between weeks 26 and 36 of gestation where initial ureteric branching and nephrons undergo massive expansion as chains of nephrons with multiple inter-connecting nephrons. Interruptions to nephrogenesis during this critical period of fetal development have a substantial impact on nephron endowment. The number of nephrons at birth impact the normal lifespan of the kidney and people with reduced nephron endowment are at increased risk of developing hypertension and chronic kidney disease.

### The role of the microbiome

In the pre-natal and post-natal periods, numerous studies are providing evidence that the microbiome plays a critical role in development and future risk of metabolic, immunologic/inflammatory, and neurodevelopmental conditions. Most of the research in this field has studied the role of intestinal microbiome in these processes, which is the focus of the following section.

In early life, the intestinal microbiota regulates immune development and homeostasis, including immune tolerance and the life-long bias of the immune Th1/Th2 response [reviewed in (45, 46)]. The critical window for the establishment of the intestinal microbiome after birth is generally held to extend to ~2 years of age, though there continues to be considerable dynamism through childhood and adolescence (47). Whether the infant microbiome is seeded *in utero* remains the subject of debate (48). Intrapartum exposure to maternal genital and intestinal microbiota during vaginal delivery further enriches the microbial communities that will ultimately colonize the newborn (49). During the post-natal period, the maternal influence over the newborn microbiome continues with breastfeeding (50). Events that disrupt these periods of maternal microbial transfer and hasten the introduction of organisms from other maternal niches (e.g., skin) or environmental sources (e.g., formula, water, food) such as PTB (51, 52), caesarian section delivery (53–55), or the early cessation of breastfeeding (56), have a significant impact on the composition of the newborn intestinal microbiome. Notably, the microbiome of both mother and fetus have been associated with maternal health status. For example, the composition of the placental microbiome has been associated with maternal stress and gestational diabetes, whereas the infant intestinal microbiome is associated with maternal obesity, maternal diet (independent of obesity), gestational diabetes, antibiotic exposure, and other environmental exposures including maternal smoking and tobacco smoke exposure [reviewed in (57)].

The composition of the intestinal microbiome in early life has been implicated in an increasing array of disease states in childhood and later life. In preterm infants, perturbations in the intestinal microbial composition precedes the development of enterocolitis (52). In other studies, the composition of the intestinal microbiome during the early “critical window” has been associated with the development of allergy and atopy (including asthma, anaphylaxis, allergic rhinitis, atopic dermatitis) as well as immune-mediated conditions such as type 1 diabetes and inflammatory bowel disease later in childhood (45, 50, 58–62).

In addition to its impact on these conditions, intestinal dysbiosis in the early life period has been associated with neurodevelopmental outcomes. The critical window for the establishment of the microbiome is aligned with periods of rapid brain development, for example, the peak of synaptic pruning in the brain coincides with the maturation of the intestinal microbiota (63–66). Synaptic pruning associated with changes in intestinal microbiota results in alterations in specific white matter structures in the brain (67). In recent studies, intestinal diversity at 1 year of age was associated with cognitive abilities at 2 (68), 3 (69), and 4 years of age (70). Intestinal microbiota have also been found to be associated with behavioral outcomes (71) and neuropsychiatric outcomes such Attention Deficit Hyperactivity Disorder (ADHD) (72, 73) and autism [reviewed in (74)].

## Promoting reproductive health to improve population health

### Current recommendations around ANC

As outlined above, investing in the promotion of perinatal care has the potential to improve outcomes far beyond acute complications associated with poor birth outcomes. An investment in widespread access to comprehensive ANC, on a global scale, has the potential to improve health outcomes for children well past the first 1,000 days of life. However, globally, only 66% of pregnant women (less in LMIC) are accessing the absolute minimum of 4 ANC visits (75). Comprehensive prenatal care generally incorporates a more rigorous visit schedule (Table 2), including a first early visit before 12 weeks' gestation, a visit every 4 weeks up until 28 weeks' gestation, a visit every 2–3 weeks from 28 to 36 weeks' gestation, and weekly visits thereafter, until delivery (76). Therefore, in high-income countries, pregnant women typically attend 7–12 routine prenatal visits across pregnancy (76, 77).

**Table 2:** Comparison of WHO antenatal care recommendations.

**Recommendation**	**Focused antenatal care (FANC)**	**Comprehensive antenatal care**
Year of implementation	2002	2015
Total visits	4	12
Minimum visits^*^	4	8
First visit	Before 12 weeks	Before 12 weeks
Visits up to 28 weeks	1 (24–26 weeks)	4–5 visits (every 4 weeks)
Visits 28–36 weeks	1 (at 32 weeks)	3–4 visits (every 2 weeks)
Visits after 36 weeks	1 (36–38 weeks)	3–4 visits (weekly)

* Minimum visits recommended for uncomplicated pregnancies. If at any time a pregnancy is judged to be high risk the schedule of care should be tailored. At the first visit, if a pregnancy is judged to be high-risk (e.g., preeclampsia; history of stillbirth or fetal growth restriction, multiple pregnancy) they are no longer eligible for the FANC and should be referred for specialized care or additional assessments.

In addition to services provided *via* basic ANC, a comprehensive approach to care includes screening for a wider array of risk factors for adverse pregnancy and long-term offspring outcomes, including perinatal anxiety, depression, and other psychosocial conditions, intimate partner violence, detailed nutritional screens and interventions, gestational diabetes, haemoglobinopathies, and a panel of high-risk infections (e.g., asymptomatic bacteriuria, syphilis, or infection with hepatitis B virus, *Chlamydia trachomatis, Neisseria gonorrhea*, cytomegalovirus, Group B streptococcus, etc.). Vaccinations and boosters including Tdap (tetanus, diphtheria, and pertussis) and influenza are also recommended as part of comprehensive ANC to prevent severe infections that could negatively impact *in utero* development and neonatal survival.

Ultrasound scans are an important component of comprehensive ANC. At the first visit (<12 weeks' gestation), ultrasound is recommended for accurate gestational dating. Accurate gestational dating is necessary to time screening and interventions, to identify and manage complications, and to inform precise birth plans that ensure safe facility deliveries (78, 79). Routine complete second trimester ultrasound scans (18–22 weeks) are used to screen for and/or diagnose fetal and placental anatomical abnormalities, identify the number of fetuses, and confirm gestational age. Furthermore, the second trimester anatomical scan can be used in conjunction with genetic screening (i.e., multiple marker serum screening) to establish risk of a chromosomal or genetic abnormality.

Inadequate ANC (defined as late or inconsistent access) increases the risk for PTB and LBW, as well as other triggers for the developmental origins of disease (i.e., missed vaccinations, and untreated maternal infections, metabolic disorders, and psychosocial conditions) in both LMIC and high-income countries (80–83).

### Reproductive health beyond prenatal care

The continuum of care for optimizing maternal-child health encompasses much more than ANC (84). The period of labor and delivery, and the first days of a newborn's life, are a high-risk period for both mother and child. To optimize maternal and child outcomes, attendance of a skilled provider at delivery, access to emergency obstetric care, and postnatal care are important to long-term maternal-child outcomes. The continuum of care for pregnant women and all women of reproductive age should also extend to sexual education, family planning (e.g., modern contraception), and safe abortion. There is abundant evidence that sexual education and family planning programs protect the *in utero* environment and reduce the burden of maternal-child morbidity and mortality by preventing acquisition of sexually transmitted infections (STIs), reducing rates of unintended pregnancies that are associated with inadequate ANC use, and decreasing the number of high-risk pregnancies more likely to end in PTB, LBW, and SGA (e.g., pregnancies in women <18 and >35 years of age, short birth intervals (<24 months), and high parity births) (84–86). Gaps in healthcare that increase the risk of poor health outcomes for the mother have the potential to translate into poor birth outcomes and could increase the risk of developmental origins of disease.

### Barriers to comprehensive antenatal and reproductive healthcare

Barriers to comprehensive ANC and reproductive care are multi-faceted and include both social and structural barriers. These barriers have been extensively studied and reviewed (87–95). In brief, they include socioeconomic, educational, and geographical obstacles (e.g., cost and distance to a health facility, misperceptions surrounding pregnancy, inability to recognize danger signs), sociocultural obstacles (e.g., stigma around pregnancy, gender norms and inequities), and negative experiences with ANC (e.g., lack of privacy, long wait times, poor quality of care and/or care providers). Evidence also suggests use of ANC increases with maternal age, education, socioeconomic status, exposure to media, and contraception use, and decreases with parity and unintended pregnancies.

While the above studies investigated barriers to care in LMIC, many of the same barriers (e.g., socioeconomic, educational, and geographical obstacles, health system barriers, negative experiences with ANC) are also reflected in high-income countries including Canada and the USA (96–99). In high-income countries, these disparities often fall along racial lines with ethnic minorities carrying a disproportionate burden of inadequate access to ANC and reproductive health (99).

Legal restrictions and intimate partner violence are two further systemic barriers to adequate reproductive health and ANC. Legal restrictions on abortion, for example, trigger barriers to maternal-fetal health in several ways. Rates of unsafe abortion are directly correlated to restrictiveness of abortion laws (100). Legal restrictions on abortion also have indirect effects on *in utero* health with implications for the developmental origins of disease, including higher rates of unintended or unwanted pregnancies that are linked with reduced utilization of ANC and higher risk of adverse birth outcomes (101). Women who experience intimate partner violence are less likely to have control over their reproductive health (i.e., access to contraception and/or protection, forced pregnancy or forced termination, etc.), less likely to have access to ANC, especially early initiation of ANC, and are at higher risk for STIs, mental health disorders, and adverse birth outcomes (86, 102–106). At the most extreme end of intimate partner violence, homicide is a leading cause of death amongst pregnant women and women of reproductive age in the US (106, 107). Finally, in addition to baseline systemic issues, acute barriers that exacerbate existing inequities in access to ANC, including humanitarian crises, armed conflict, and global events (e.g., COVID-19 pandemic), must also be acknowledged for their impact on the *in utero* environment.

### Where do we go from here?

Many policies focus on individual aspects of maternal-child healthcare, rather than the continuum of care necessary for long-term positive pregnancy outcomes. Approximately 92% of all pregnancies occur in LMIC, where barriers to reproductive health are frequent, and access to ANC is most limited (101). ANC policies in many LMIC have incorporated versions of the WHO's four-visit FANC model, which has since been proven inadequate (9). In sub-Saharan Africa, only 7% of pregnant women attend the currently recommended 8+ ANC visits (93). National policies must be updated according to evidence-based recommendations. There are also disconnects between policy existence and implementation. Effective policy implementation requires underlying shifts in attitudes and behavior. Even with national ANC policies in place, unmet need for ANC remains overwhelming: in LMIC, an estimated 50 million pregnant women attend <4 ANC visits, 31 million do not deliver at a health facility, and 133 million do not receive the treatments needed for common STIs (108).

There has been substantial progress in improving maternal-child health over the past decade. Despite growing evidence highlighting the long-term, intergenerational impacts of the *in utero* environment, current socio-political indications in some regions of the world threaten a slide toward deprioritizing women's health. UNICEF reports that half of all maternal deaths and over half of all neonatal deaths are directly attributable to poor quality ANC (109). There is an urgent need to reinforce the developmental origins of disease and shift policy toward the prioritization of maternal-child health in global action and policies.

The most straightforward strategy to improve maternal-child health is investment. While standardized policy is required to guide global progress, policy change will be ineffective without simultaneously investing in strengthening overall health systems. Investment in quality of ANC health services (e.g., increasing health center staff, specialized personnel, and infrastructure; equitable distribution of medical commodities including medicines, vaccines, and technologies, etc.), infrastructure critical to accessing health services (e.g., roads connecting rural and urban communities, affordable transportation, etc.), and governmental investments to promote utilization (e.g., free maternal healthcare, primary resource allocation) could remove barriers to access for pregnant women (88, 110). The Guttmacher institute estimates that increasing annual investment in LMICs by $4.80 per capita would close existing gaps in sexual and reproductive health services (108). Increasing investment in the full continuum of reproductive health care would also have significant cascading impacts—for example, for every additional dollar invested in contraceptive services, an estimated three dollars in costs from maternal, neonatal, and abortion care would be saved, by averting unintended pregnancies (108).

Advocacy and community engagement are cornerstones to the behavior and attitude changes required to support policy. Women's empowerment and access to information/education directly correlate with early and consistent utilization of ANC and access to important pregnancy interventions (90, 93, 95), and promoting gender equity in both healthcare seekers and healthcare providers increases utilization of ANC (95, 111).

Finally, to enact effective policy change, we need to optimize the evidence base used to inform decision-making. There exist many gaps in our full scope of understanding of the biological, policy, and implementation issues facing maternal-child health, and even more gaps in how to solve them (112–117). Continued data collection to address these neglected areas, quality interventional research, and monitoring and evaluation of policies and implementation to allow for contextual adaptations, are critical to informing evidence-based approaches to improving immediate and long-term maternal-child health. We must continue to promote and support research in the field that fosters innovative, scalable, and empowering strategies to fill existing gaps in care [e.g., self-care interventions for provision of sexual and reproductive health in humanitarian crisis settings (117); integrated packages of care covering the full reproductive health continuum (84); interventions that acknowledge and improve maternal mental health (27), etc.].

## Conclusion

Despite well-characterized barriers to ANC and allied healthcare, a devastating number of maternal-child dyads do not receive adequate, if any, ANC. Most studies highlighting provision of ANC and barriers to access have focused on its impacts on maternal and neonatal mortality. In this review, we show that mortality is only the tip of the iceberg. If we do not prioritize reproductive health and ANC, millions more children will experience preventable, lifelong, and intergenerational morbidities including chronic kidney disease and neurocognitive deficits that are triggered by preventable causes *in utero*. Current global maternal health policies and attitudes are failing our most vulnerable populations.

The evidence on the developmental origins of many chronic diseases support investments in maternal health to achieve broad impacts on population health. Most data on the developmental origins of health and disease come from high-income settings where there are differences in maternal health and nutritional status prior to and during pregnancy and differences in the nature and frequency of infectious exposures during pregnancy. An estimated 90% of global pregnancies occur in LMIC with an estimated 125 million pregnancies at risk of malaria annually (118). Here, we discuss developmental origins of disease and the rationale this provides for expanded ANC and allied reproductive and pediatric healthcare, without a principal focus on LMIC. However, we recognize that the burden of developmental origins of disease occurs principally in resource constrained settings, which justify enhanced attention and resource allocation (1, 2).

We know that investment in women and children living in poverty show enormous returns. In the face of conclusive evidence that comprehensive ANC improves health outcomes for mother and child, far too many women are not receiving adequate ANC. While the principal focus is and should remain on acute outcomes for mother and child, we propose that we can no longer ignore the vital importance of the *in utero* environment on the lifelong health and development of every child. Perhaps enhancing the focus on developmental origins of disease will further strengthen the evidence base required to shift focus and resources to reduce barriers and expand services of ANC. Protecting pregnant women and the *in utero* environment has the potential to promote healthy intergenerational outcomes.

## Data availability statement

The original contributions presented in the study are included in the article/supplementary material, further inquiries can be directed to the corresponding author/s.

## Author contributions

All authors listed have made a substantial, direct, and intellectual contribution to the work and approved it for publication.

## Funding

This work was supported by the Canadian Institutes of Health Research Foundation (grant no. FDN-148439 to KK), the Canada Research Chair Program (to KK), Open Philanthropy (to KK), and donations from Kim Kertland (to KK).

## Conflict of interest

The authors declare that the research was conducted in the absence of any commercial or financial relationships that could be construed as a potential conflict of interest.

## Publisher's note

All claims expressed in this article are solely those of the authors and do not necessarily represent those of their affiliated organizations, or those of the publisher, the editors and the reviewers. Any product that may be evaluated in this article, or claim that may be made by its manufacturer, is not guaranteed or endorsed by the publisher.
